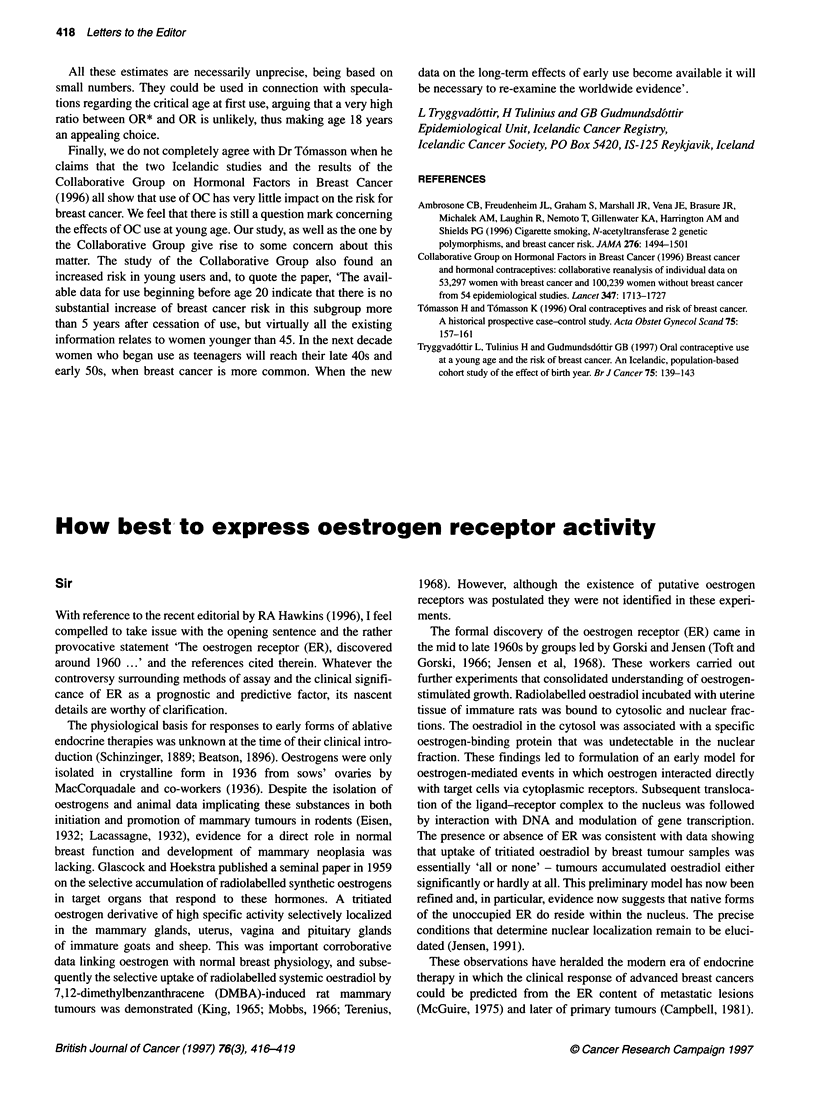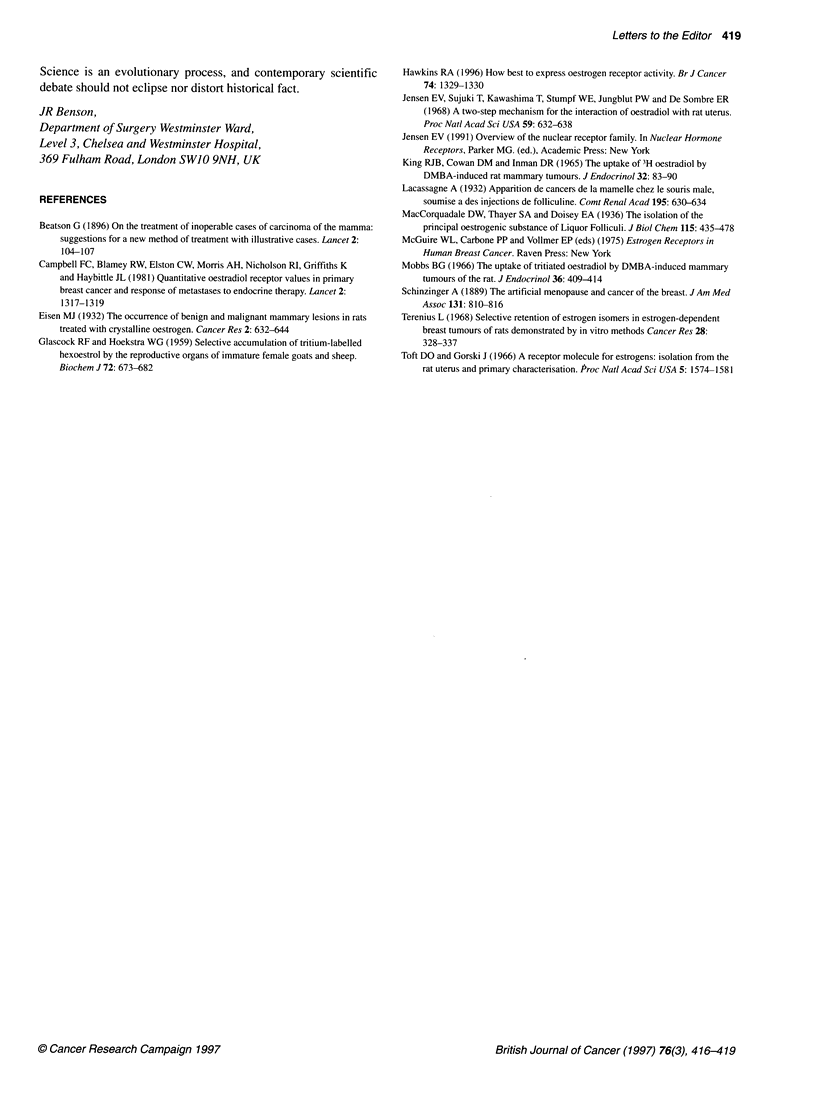# How best to express oestrogen receptor activity.

**DOI:** 10.1038/bjc.1997.402

**Published:** 1997

**Authors:** J. R. Benson


### How best to express oestrogen receptor activity

**Sir**

With reference to the recent editorial by RA Hawkins (1996), I feel compelled to take issue with the opening sentence and the rather provocative statement 'The oestrogen receptor (ER), discovered around 1960 ...' and the references cited therein. Whatever the controversy surrounding methods of assay and the clinical significance of ER as a prognostic and predictive factor, its nascent details are worthy of clarification.

The physiological basis for responses to early forms of ablative endocrine therapies was unknown at the time of their clinical introduction (Schinzinger, 1889; Beatson, 1896). Oestrogens were only isolated in crystalline form in 1936 from sows' ovaries by MacCorquadale and co-workers (1936). Despite the isolation of oestrogens and animal data implicating these substances in both initiation and promotion of mammary tumours in rodents (Eisen, 1932; Lacassagne, 1932), evidence for a direct role in normal breast function and development of mammary neoplasia was lacking. Glascock and Hoekstra published a seminal paper in 1959 on the selective accumulation of radiolabelled synthetic oestrogens in target organs that respond to these hormones. A tritiated oestrogen derivative of high specific activity selectively localized in the mammary glands, uterus, vagina and pituitary glands of immature goats and sheep. This was important corroborative data linking oestrogen with normal breast physiology, and subsequently the selective uptake of radiolabelled systemic oestradiol by 7,12-dimethylbenzanthracene (DMBA)-induced rat mammary tumours was demonstrated (King, 1965; Mobbs, 1966; Terenius, 1968). However, although the existence of putative oestrogen receptors was postulated they were not identified in these experiments.

The formal discovery of the oestrogen receptor (ER) came in the mid to late 1960s by groups led by Gorski and Jensen (Toft and Gorski, 1966; Jensen et al, 1968). These workers carried out further experiments that consolidated understanding of oestrogen-stimulated growth. Radiolabelled oestradiol incubated with uterine tissue of immature rats was bound to cytosolic and nuclear fractions. The oestradiol in the cytosol was associated with a specific oestrogen-binding protein that was undetectable in the nuclear fraction. These findings led to formulation of an early model for oestrogen-mediated events in which oestrogen interacted directly with target cells via cytoplasmic receptors. Subsequent translocation of the ligand–receptor complex to the nucleus was followed by interaction with DNA and modulation of gene transcription. The presence or absence of ER was consistent with data showing that uptake of tritiated oestradiol by breast tumour samples was essentially 'all or none' – tumours accumulated oestradiol either significantly or hardly at all. This preliminary model has now been refined and, in particular, evidence now suggests that native forms of the unoccupied ER do reside within the nucleus. The precise conditions that determine nuclear localization remain to be elucidated (Jensen, 1991).

These observations have heralded the modern era of endocrine therapy in which the clinical response of advanced breast cancers could be predicted from the ER content of metastatic lesions (McGuire, 1975) and later of primary tumours (Campbell, 1981).

© Cancer Research Campaign 1997

Science is an evolutionary process, and contemporary scientific debate should not eclipse nor distort historical fact.

*JR Benson,*
*Department of Surgery Westminster Ward,*
*Level 3, Chelsea and Westminster Hospital,*
*369 Fulham Road, London SW10 9NH, UK*

#### REFERENCES

Beatson G (1896) On the treatment of inoperable cases of carcinoma of the mamma: suggestions for a new method of treatment with illustrative cases. *Lancet* **2**: 104–107

Campbell FC, Blamey RW, Elston CW, Morris AH, Nicholson RI, Griffiths K and Haybittle JL (1981) Quantitative oestradiol receptor values in primary breast cancer and response of metastases to endocrine therapy. *Lancet* **2**: 1317–1319

Eisen MJ (1932) The occurrence of benign and malignant mammary lesions in rats treated with crystalline oestrogen. *Cancer Res* **2**: 632–644

Glascock RF and Hoekstra WG (1959) Selective accumulation of tritium-labelled hexoestrol by the reproductive organs of immature female goats and sheep. *Biochem J* **72**: 673–682

Hawkins RA (1996) How best to express oestrogen receptor activity. *Br J Cancer* **74**: 1329–1330

Jensen EV, Sujuki T, Kawashima T, Stumpf WE, Jungblut PW and De Sombre ER (1968) A two-step mechanism for the interaction of oestradiol with rat uterus. *Proc Natl Acad Sci USA* **59**: 632–638

Jensen EV (1991) Overview of the nuclear receptor family. In *Nuclear Hormone Receptors*, Parker MG. (ed.), Academic Press: New York

King RJB, Cowan DM and Inman DR (1965) The uptake of $^{3}$H oestradiol by DMBA-induced rat mammary tumours. *J Endocrinol* **32**: 83–90

Lacassagne A (1932) Apparition de cancers de la mamelle chez le souris male, soumise a des injections de folliculine. *Comt Renal Acad* **195**: 630–634

MacCorquadale DW, Thayer SA and Doisey EA (1936) The isolation of the principal oestrogenic substance of Liquor Folliculi. *J Biol Chem* **115**: 435–478

McGuire WL, Carbone PP and Vollmer EP (eds) (1975) *Estrogen Receptors in Human Breast Cancer*. Raven Press: New York

Mobbs BG (1966) The uptake of tritiated oestradiol by DMBA-induced mammary tumours of the rat. *J Endocrinol* **36**: 409–414

Schinzinger A (1889) The artificial menopause and cancer of the breast. *J Am Med Assoc* **131**: 810–816

Terenius L (1968) Selective retention of estrogen isomers in estrogen-dependent breast tumours of rats demonstrated by in vitro methods *Cancer Res* **28**: 328–337

Toft DO and Gorski J (1966) A receptor molecule for estrogens: isolation from the rat uterus and primary characterisation. *Proc Natl Acad Sci USA* **5**: 1574–1581

© Cancer Research Campaign 1997